# The myositis clinical phenotype associated with anti-Zo autoantibodies: a case series of nine UK patients

**DOI:** 10.1093/rheumatology/kez504

**Published:** 2019-10-26

**Authors:** Sarah L Tansley, Zoe Betteridge, Hui Lu, Emma Davies, Simon Rothwell, Paul P New, Hector Chinoy, Patrick Gordon, Harsha Gunawardena, Mark Lloyd, Richard Stratton, Robert Cooper, Neil J McHugh

**Affiliations:** 1 Department of Pharmacy and Pharmacology, The University of Bath, Bath; 2 Department of Rheumatology, North Bristol NHS Trust, Bristol; 3 Centre for Musculoskeletal Research, Faculty of Biology, Medicine and Health, The University of Manchester, Manchester; 4 MRC/ARUK Centre for Integrated Research into Musculoskeletal Ageing, University of Liverpool, Liverpool; 5 National Institute for Health Research Manchester Biomedical Research Centre, Manchester University NHS Foundation Trust, The University of Manchester, Manchester; 6 Department of Rheumatology, Salford Royal NHS Foundation Trust, Manchester Academic Health Science Centre, Salford; 7 Department of Rheumatology, Kings College, London; 8 Department of Rheumatology, Frimley Park NHS Foundation Trust, Surrey; 9 UCL Division of Medicine, Centre for Rheumatology and Connective Tissue Diseases, London, UK

**Keywords:** myositis and muscle disease, autoantigens and autoantibodies, respiratory, biomarkers

## Abstract

**Objectives:**

It has been over 10 years since the first report of autoantibodies directed against phenylalanyl tRNA synthetase (anti-Zo) in a patient with features of the anti-synthetase syndrome. In that time no further cases have been published. Here we aim to characterize more fully the clinical phenotype of anti-Zo–associated myositis by describing the clinical features of nine patients.

**Methods:**

Anti-Zo was identified by protein-immunoprecipitation in patients referred for extended spectrum myositis autoantibody testing at our laboratory. Results were confirmed by immunodepletion using a reference serum. Medical records were retrospectively reviewed to provide detailed information of the associated clinical phenotype for all identified patients. Where possible, HLA genotype was imputed using Illumina protocols.

**Results:**

Nine patients with anti-Zo were identified. The median age at disease onset was 51 years, and six patients were female. Seven patients had evidence of inflammatory muscle disease, seven of interstitial lung disease and six of arthritis. The reported pattern of interstitial lung disease varied with usual interstitial pneumonia, non-specific interstitial pneumonia and organizing pneumonia all described. Other features of the anti-synthetase syndrome such as RP and mechanics hands were common. HLA data was available for three patients, all of whom had at least one copy of the HLA 8.1 ancestral haplotype.

**Conclusion:**

Patients with anti-Zo presenting with features of the anti-synthetase syndrome and interstitial lung disease is a common finding. Like other myositis autoantibodies, there is likely to be a genetic association with the HLA 8.1 ancestral haplotype.

**Rheumatology key messages**

- Patients with anti-Zo autoantibodies have features of the anti-synthetase syndrome.
- Interstitial lung disease is common in patients with anti-Zo autoantibodies.
- An association with the HLA8.1 ancestral haplotype is suggested.

## Introduction

The anti-synthetase syndrome is a well-described clinical syndrome consisting of myositis, interstitial lung disease (ILD), non-erosive arthritis, RP, fever, and characteristic skin changes termed ‘mechanics’ hands’ [1]. Anti-synthetase syndrome is considered to be a subgroup of the idiopathic inflammatory myopathies (IIMs), although incomplete versions of the syndrome are well recognized and not all patients have muscle involvement. No formal definition or classification criteria for anti-synthetase syndrome has been widely accepted, but the development of EULAR-ACR Classification Criteria for Antisynthetase Syndrome is an ongoing initiative.

Patients with anti-synthetase syndrome have autoantibodies directed against tRNA synthetases, a family of cytoplasmic enzymes responsible for catalysing the binding of amino acids to their corresponding tRNAs [1]. There are 20 different tRNA synthetases corresponding to the 20 different amino acids, and thus far autoantibodies targeting eight have been described in patients with IIM. Anti-Jo-1, targeting histidyl tRNA synthetase, is the most common autoantibody in adults with IIM and can be identified in 15–30% of patients [1, 2]. The remaining anti-tRNA synthetases; anti-PL7 (threonyl), anti-PL12 (alanyl), anti-OJ (isoleucyl), anti-KS (asparginyl), anti-EJ (glycyl), anti-Zo (phenylalanyl) and anti-Ha (tyrosyl) are rarer, collectively occurring in 10–20% of cases [1, 2]. While anti-synthetase syndrome is generally viewed as one syndrome, there are established differences between the clinical associations of the different anti-synthetase autoantibodies [3–9]. While muscle disease is common in patients with anti-Jo1, anti-PL-7 or anti-EJ, those with anti-PL-12, anti-KS or anti-OJ in contrast often have lung-dominant disease [9]. We were the first to report autoantibodies directed against phenylalanyl tRNA synthetase (anti-Zo) in a single patient with myositis and now report on a series of nine patients with this autoantibody [10].

## Methods

### Patients

Our laboratory has to date analysed >3000 serum samples by immunoprecipitation [2, 11]. Patients included in this series were identified through autoantibody analysis in our laboratory for research or diagnostic purposes. Seven patients included were enrolled in the UKMyoNet cohort. This cohort includes patients aged 18 years of age or older who fulfill the Bohan and Peter criteria for probable or definite PM/DM. A standardized clinical data collection form, detailing demographics and individual clinical details is used. Collaborating physicians at each study site confirm the presence of ILD (by pulmonary function testing and thoracic imaging) and cancer-associated myositis (in the opinion of the recruiting physician, by relevant investigations). Collection of blood from patients was undertaken under the regulations of the local research ethics committees [12, 13]. A further two patients were identified through the diagnostic screening service, samples having been sent for specialist, extended-spectrum autoantibody testing. These patients were not required to meet any diagnostic criteria.

Case notes were retrospectively reviewed for all patients identified as anti-Zo–positive. There was no requirement for patients to be screened by thoracic imaging or pulmonary function testing, nor for formal muscle strength testing or muscle biopsy to be performed. All investigations were arranged by the treating physician as deemed necessary. ILD was diagnosed by the treating physician on the basis of investigations arranged locally. Muscle involvement was similarly diagnosed on the basis of examination findings and local investigations.

Written consent to participate and provide biological samples was obtained from all subjects according to the Declaration of Helsinki, and in compliance with local ethics committee regulations.

### Autoantibody identification

All samples were screened for the presence of autoantibodies by radiolabelled protein immunoprecipitation as described previously [10]. Where samples immunoprecipitated 58 kDa and 68 kDa bands, the presence of anti-Zo was confirmed by immunodepletion using a reference serum (case 1, previously confirmed positive for anti-Zo by mass spectrometry [10]).

For those patients participating in the UKMyonet cohort, samples were taken at the time of enrolment, which may have been several years after disease onset. For patients identified as having anti-Zo autoantibodies, the samples analysed were taken up to 8 years post disease onset.

### Indirect immunofluorescence

Indirect immunofluorescence was performed on HEp-2 cells (Nova-lite, Inova) according to the manufacturers’ instructions. All slides were read blindly and independently by two independent observers.

### Evaluation of ENAs

The additional presence of antibodies targeting Ro52 and other ENAs was determined using line blot (ANA profile 5, Euroimmun) according to the manufacturer’s instructions.

### HLA genotyping

Classical HLA alleles were imputed using SNP2HLA from Immunochip genotyping data using a reference panel generated by the Type 1 Diabetes Genetics Consortium [14]. Genotyping was performed in accordance with Illumina protocols at the Centre for Musculoskeletal Research, University of Manchester, UK. Standard quality control was performed as described previously [15].

## Results

**Fig. 1:**
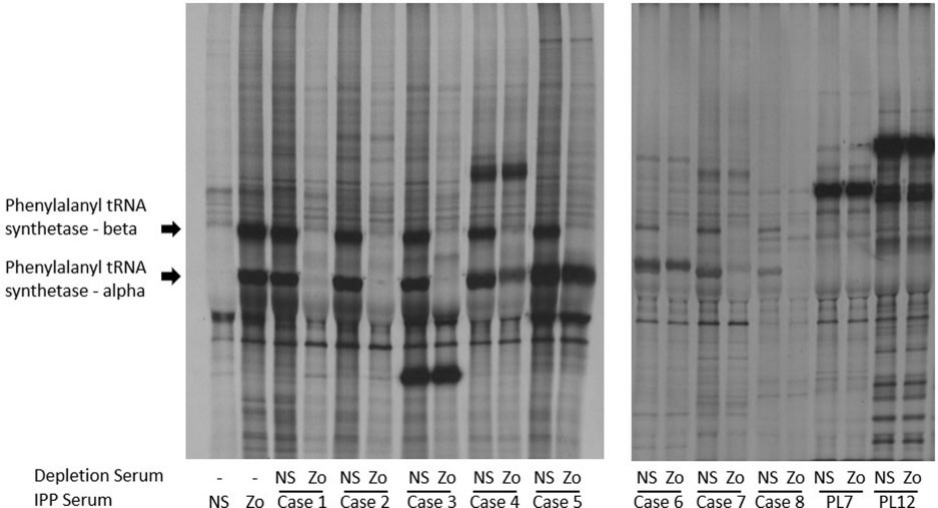
Immunodepletion experiments using prototype serum confirmed the presence of anti-Zo in all samples studied Autoradiographs of 9% SDS–PAGE of immunoprecipitates from cases 1–8 serum, normal control serum or PL12/PL7 positive control sera immunoprecipitated using either control [^35^S] methionine-labelled cell extract (–), or [^35^S] methionine-labelled cell extract depleted with either normal sera (NS) or case 1 sera (anti-phenylalanyl tRNA synthetase previously confirmed using mass spectrometry [10]). The bands corresponding to phenylalanyl tRNA synthetase alpha (55 kDa) and phenylalanyl tRNA synthetase alpha (65 kDa) are indicated.

Nine patients were identified as having 58 kDa and 68 kDa bands on immunoprecipitation, and all were subsequently confirmed to have autoantibodies targeting phenylalanyl tRNA synthetase by immunodepletion, (see Fig. 1). All had a fine cytoplasmic speckled pattern on immunofluorescence, with titres ranging between 1:160 and 1:1280. Anti-Zo did not occur in conjunction with any other myositis-specific autoantibody, but a number of cases had additional autoantibodies targeting ENA, in particular Ro52, see Table 1.

**Table 1:** Clinical and laboratory findings in patients with anti-Zo autoantibodies

	Case 1	Case 2	Case 3	Case 4	Case 5	Case 6	Case 7	Case 8	Case 9
Demographics									
Gender	Female	Female	Female	Female	Male	Female	Male	Male	Female
Ethnicity	Caucasian	Caucasian	Afro-Caribbean	African	Caucasian	Caucasian	Caucasian	Caucasian	Caucasian
Age at onset	49	35	62	40	35	52	40	51	79
Muscle disease									
Clinical	Proximal myopathy	Proximal myopathy	Myalgia	Proximal myopathy	Myalgia. Mild proximal myopathy	Proximal myopathy	Not present	Myalgia	Not present
Muscle enzymes	CK >9000	CK >1000	CK elevated	CK elevated	CK >2000	CK elevated	Mild CK rise. 255 maximum	CK >1500.	Normal
EMG	Not done	Normal	Abnormal	Myopathic	Myopathic	Normal	Not done	Not done	Not done
Muscle biopsy	Necrotizing myopathy	HLA class 1 upregulation	Not done	Inflammatory myopathy	Not done	Inflammatory myopathy	Not done	Not done	Not done
Other	Not applicable	MRI showed fasciitis	Not applicable	Not applicable	Not applicable	Not applicable	Not applicable	Oesophageal dysmotility on barium swallow	Not applicable
Interstitial lung disease									
	Yes (NSIP)	Yes (NSIP)	Yes (UIP)	Not present	Yes (OP with mild NSIP )	Not present	Yes (NSIP)	Yes (OP)	Yes (NSIP)
Other anti-synthetase syndrome features									
*RP*	Yes	Yes	Yes	Yes	Yes	Yes	Yes	Not present	Yes
*Arthritis/arthralgia*	Yes	Yes	Yes	Not present	Yes	Yes	Not present	Not present	Yes
*Mechanics’ hands*	Not present	Yes	Not present	Not present	Yes	Not available	Yes	Yes	Not present
*Rash*	Not present	Not present	Not present	Heliotrope rash and V-sign	Gottron’s papules	Not present	Heliotrope rash	Not present	Not present
Laboratory findings									
*Hep2 Indirect Immunofluorescence*	ANA negative	ANA negative	ANA negative	ANA negative	Homogeneous ANA, Fine Cytoplasmic Speckle 1: 640	ANA negative	ANA negative	ANA negative	ANA negative
	Fine Cytoplasmic Speckle 1: 1280	Fine Cytoplasmic Speckle 1: 1280	Fine Cytoplasmic Speckle 1: 640	Fine Cytoplasmic Speckle 1: 160		Fine Cytoplasmic Speckle 1: 160	Fine Cytoplasmic Speckle 1: 1280	Fine Cytoplasmic Speckle 1: 320	Fine Cytoplasmic Speckle 1: 1280
*ENA*	Ro52	Ro52	Ro52, SSB	Ro52, SSA	Ro52, SSA, SSB, anti-nucleosome	Ro52, SSA	Negative	Negative	Negative
*Genotype*	HLA-DRB1*03: 01 homozygous	HLA-DRB1*03: 01 heterozygous	Not available	Not available	Not available	HLA-DRB1*03: 01 heterozygous	Not available	Not available	Not available
The effect of autoantibody status on ACR/EULAR Classification Criteria for Idiopathic Inflammatory Myopathies [16]									
*Probability of IIM*	39–96%	23–91%	13–83%	97–100% Definite IIM	70–99% Probable IIM	39–96%	47–97%	23–83%	4–57%
*Probability of IIM including anti-Zo* ^a^	97–100%	94–100%	88–100%	100%	99–100%	97–100% Definite IIM	98-100%	94–100%	66–98%
	Definite IIM	Definite IIM	Probable IIM	Definite IIM	Definite IIM		Definite IIM	Definite IIM	Definite IIM

The demographics and clinical features of patients identified as having anti-Zo autoantibodies are summarized below along with additional laboratory findings. ^a^Anti-Jo1 selected in place of anti-Zo. CK: creatine kinase; IIM: idiopathic inflammatory myophathy; NSIP: non-specific interstitial pneumonia; UIP: usual interstitial pneumonia; OP: organizing pneumonia. Interstitial lung disease patterns as reported on high-resolution CT.

HLA data was available for three patients, all of whom were Caucasian, and all of whom had at least one copy of the ancestral haplotype DR3-DQ2.

Patients with anti-Zo autoantibodies had a median age of disease onset of 51 years and a female to male ratio of 6:3. All patients had many features of the anti-synthetase syndrome, including inflammatory arthritis, RP and mechanics’ hands. DM-associated skin changes were present in three patients. Both muscle involvement and ILD were common, each occurring in seven (78%) of patients. Just over half of the patients had both muscle and lung disease. The pattern of ILD reported on high-resolution CT varied and where identified ILD was present at diagnosis. Patient nine had a history of endometrial carcinoma, but no other patients had a history of concurrent malignancy. The clinical features of each patient are summarized in Table 1.

Using the available data, the probability of IIM as the diagnosis was calculated using the recently published ACR/EULAR classification criteria calculator (http://www.imm.ki.se/biostatistics/calculators/iim/) [16]. Results ranged from 4–21% (patient 9) to 97–100% (patient 4). The inclusion of anti-Jo1 as a surrogate for anti-Zo increased the probability of IIM to ‘probable’ for patients 3 and 9 and to ‘definite’ for the remaining 7 patients. Probability scores for all patients can be found in Table 1.

Data on treatment and outcome was limited. All patients received steroids in addition to further immunosuppressive agents, as is standard clinical practice in the UK. Four patients received i.v. CYC alongside prednisolone, and all responded well initially. One patient required a second course of CYC following a relapse 2 years later, and another patient relapsed 6 months later and subsequently received treatment with rituximab. Patients were maintained on a number of different immunosuppressive agents, including AZA, MMF, CSA and tacrolimus. At the time of writing, one patient had remained well off all treatment for 2 years.

## Discussion

Anti-Zo is a rare anti-synthetase autoantibody, and this case series is to date the largest cohort of such patients described. All patients had a cytoplasmic speckle on indirect Hep2 cell immunofluorescence. The proportion of females (66.7%) was similar to the 69.6% previously reported for European patients with IIM [2]. Similarly, the median age at onset of 50 years was similar to the 51 years previously reported for European patients with IIM, although we note that six of the nine patients were aged 51 years or less [2]. The frequency of different myositis-related autoantibodies is known to vary depending on age at disease onset, and it is possible that anti-Zo is more common in younger adults. While IIM can be associated with malignancy, the anti-synthetase syndrome is not generally believed to carry a significant increased risk of associated malignancy [2]. The only patient with malignancy in this series was aged 79 years old, and no clear link with anti-Zo is proposed.

ILD is a major cause of mortality in myositis and occurs in up to 90% of adults with anti-synthetase syndrome [17, 18]. Patients with non-Jo-1 anti-synthetase autoantibodies have been found to have a worse survival than those with anti-Jo-1 [3]. This may reflect that these patients are more likely to present with incomplete anti-synthetase syndrome, without muscle involvement, in addition to challenges in autoantibody identification and diagnosis. Only two patients with anti-Zo were classified as probable or definite IIM using the recently ACR/EULAR Classification Criteria for Idiopathic Inflammatory Myopathies [16]. ILD was common in patients with anti-Zo: Seven (78%) of patients were identified as having ILD, although the pattern of ILD reported on high-resolution CT varied. It should be noted that the reporting of HRCTs was not standardized and is presented as was reported by the reporting radiologist at each patient’s base hospital.

It is noteworthy that the sera from two-thirds of our patients with anti-Zo also contained autoantibodies targeting anti-Ro52. Anti-Ro52 has been previously shown to occur commonly alongside other anti-synthetase autoantibodies and has been associated with more severe ILD [19, 20].

HLA variants on chromosome 6 have been established as the strongest genetic risk factors for the development of myositis [15]. Associations with certain HLA alleles have been reported for various myositis-specific autoantibodies, and the most well established is between anti-Jo-1 and the ancestral haplotype containing HLA-DRB1*03:01 [21]. Although data was limited and available from just three patients in this series, it is noteworthy that all contained at least one copy of the HLA 8.1 ancestral haplotype, which includes HLA-DRB1*03:01. Further work is needed, but as for other myositis-specific autoantibodies, patients with anti-Zo are likely to have independent associations with the HLA 8.1 ancestral haplotype, which may relate to peptide-binding affinity.

All but two patients in this series had evidence of muscle involvement, but given that all patients were referred for myositis autoantibody testing and seven were enrolled in the UKMyonet cohort and fulfilled Bohan and Peter diagnostic criteria, the high proportion of muscle disease is perhaps not surprising. Some anti-synthetase autoantibody phenotypes are associated with ‘lung-dominant’ disease’, and anti-synthetase autoantibodies have been reported in patients diagnosed with idiopathic ILD. Patients with anti-Zo–associated anti-synthetase syndrome do not all have evidence of muscle involvement, and this should be considered in patients presenting with ILD and/or with other features of the anti-synthetase syndrome, such as arthritis and RP, particularly when a cytoplasmic speckle is seen on indirect Hep2 cell immunofluorescence.

In conclusion, patients with anti-Zo autoantibodies present with features of the anti-synthetase syndrome. Most patients have myositis and ILD. Where anti-Zo autoantibody testing is not available, a cytoplasmic speckle on indirect immunofluorescence of Hep2 cells and the presence of anti-Ro52 could provide useful clues. As with other myositis-specific autoantibodies, there is likely to be an association with the HLA 8.1 ancestral haplotype.

*Funding:* This work was funded by the Bath Institute of Rheumatic Diseases. This study includes funding from the Medical Research Council (MR/N003322/1) and Arthritis Research UK Programme Grant 18474. This report includes independent research supported by the NIHR Biomedical Research Centre Funding Scheme. The views expressed in this publication are those of the authors and not necessarily those of the NHS, the National Institute for Health Research or the Department of Health.

*Disclosure statement:* The authors have declared no conflicts of interest.
